# Telemedicine experience in the Brazilian Amazon

**Published:** 2012-06-15

**Authors:** Christopher Robert Jones, Ricardo Bertoglio Cardoso, Helena Willhelm Oliveira, Maria Helena Itaqui Lopes, Edison Huttner, Eder Huttner, Mario Wiehe, Sergio Antonio Curcio Celia2, Thais Russomano

**Affiliations:** Telemedicine Laboratory, Microgravity Centre, PUCRS, Brazil; Telemedicine Laboratory, Microgravity Centre, PUCRS, Brazil; Telemedicine Laboratory, Microgravity Centre, PUCRS, Brazil; School of Medicine, PUCRS, Brazil; Nucleus of Indigenous Culture Study and Research – PUCRS, Brazil; Nucleus of Indigenous Culture Study and Research – PUCRS, Brazil; Sao Lucas Hospital, PUCRS, Brazil; Telemedicine Laboratory, Microgravity Centre – PUCRS, Brazil; Telemedicine Laboratory, Microgravity Centre – PUCRS, Brazil

**Keywords:** telecardiology, teledermatology, PUCRS, Brazilian Amazon, telemedicine experience

## Abstract

**Introduction:**

The development of telecommunication technologies and the diffusion of its eHealth applicability have enabled the implementation of a wide range of telemedicine systems, supporting clinical practices in different regions of the world. Distant and poorer areas of the globe, often characterized by difficult access and hazardous environments, are those that can most benefit from availability of remote consultations. This is due to the high costs associated with the transportation of specialized health teams and medical equipment between major cities and small villages, sometimes essential for the provision of adequate health care. Brazil is a country of continental dimensions with socioeconomic inequalities and uneven distribution of specialized health care, making it an ideal environment for establishment of eHealth initiatives.

**Objective:**

The main objectives were (1) to develop an efficient method for acquiring and delivering patient medical information in remote areas using local Internet and (2) to assist urban and Indian populations in the Brazilian Amazon presenting with skin lesions and cardiovascular diseases.

**Methodology:**

Ethical approval was granted by the research and Ethics Committees of PUCRS, Porto Alegre, Brazil. The project comprised: (1) triage and patient interview; (2) digital ECG and skin picture acquisition; (3) data management, storage and transmission; (4) delivery of expert second opinion based on development of a secure and confidential communication system between the receiving and delivering sites. Upon internet availability, the system was programmed to connect to the Microgravity Centre’s main server and to synchronise all collected data. Specialist health professionals accessed this server and the electronic patient records via a secure application installed on personal computers. Patient information, images, and exams were analysed by health specialists at the delivering site, and an opinion and treatment suggestion was offered. Cases that proved inconclusive were due to a lack of available information or the need for further laboratory exams. The opinions were secured in an encrypted PDF envelope and returned to the receiving site, where final diagnosis and treatment remained the responsibility of the local health team.

**Results:**

Telecardiology (n=98): 59 (60.2%) normal ECGs, whilst 39 (39.8%) were altered, showing arrhythmias, conductive alterations and signs of ventricular hypertrophy. No patients presented signs of acute ischemia. In teledermatology (n=110): 57 (51.8%) patients did not present any skin disease. Among the patients with dermatological problems (n=53; 48.2%), the most common diagnoses were eczema, pityriasis versicolor, tinea, onychomycosis, and superficial mycosis. In five patients, the dermatologist suspected a cancerous lesion and recommended further investigation.

**Conclusion:**

The telemedicine tools and the telecommunication system developed for this project proved to have a great applicability for diagnosis of dermatological skin conditions and for assessment of patient cardiovascular diseases. It was possible to remotely diagnose dermatological and cardiovascular conditions in a short period of time, at low cost and without the need for transportation of patients to other locations. It is believed that the eHealth assistance model applied in this project can be transferred to other places that have access to an Internet connection.

